# Identification of B-Cell Linear Epitopes on pE146L Protein of African Swine Fever Virus Using Monoclonal Antibodies

**DOI:** 10.3390/microorganisms14081707

**Published:** 2026-08-04

**Authors:** Aiping Wang, Yadi Yuan, Haili Wang, Wenying Yan, Xiao Liu, Yanwei Wang, Yanhua Qi, Yumei Chen, Xifang Zhu, Gaiping Zhang

**Affiliations:** 1 Longhu Laboratory of Advanced Immunology, Zhengzhou 450046, China; 2 School of Life Sciences, Zhengzhou University, Zhengzhou 450001, China; 3 Henan Provincial Key Laboratory of Immunobiology, Zhengzhou 450001, China

**Keywords:** African swine fever virus, pE146L, mAbs, epitopes

## Abstract

African swine fever virus (ASFV) is a highly pathogenic DNA virus that continues to circulate globally and poses a serious threat to the swine industry. However, the antigenic complexity of ASFV poses formidable challenges for vaccine and diagnostic development. The pE146L protein, which serves as a structural component of the inner envelope, is vitally required for efficient ASFV replication. In this study, the pE146L protein was expressed in *E. coli* Rosetta (DE3) cells, and five monoclonal antibodies (mAbs) were generated by immunizing BALB/c mice with the purified pE146L. Epitope fine mapping using a panel of truncated overlapping fragments identified five B-cell linear epitopes: aa37–45, aa65–80, aa86–94, aa86–101, and aa115–124, recognized by mAbs 8F3, 1B8, 5C10, 10F4, and 4B7, respectively. Furthermore, synthesized peptides SP1–SP5 were designed based on these sequences, wherein SP5 was prepared as an extended peptide spanning aa110–124 to potentially enhance antibody binding or peptide presentation. These synthetic peptides reacted not only with their respective mAbs but also with ASFV-positive pig sera, among which SP3 exhibited the strongest immunoreactivity. Sequence alignment revealed that all five epitopes were highly conserved among ASFV genotypes I, II, and the emerging I/II recombinant strains. Collectively, these findings identify novel antigenic epitopes of pE146L, providing preliminary candidates that may contribute to the future development of ASFV serological diagnostics.

## 1. Introduction

African swine fever caused by African swine fever virus (ASFV) is a highly transmissible hemorrhagic disease affecting domestic and wild pigs, with a mortality rate of up to 100% [1]. Soft ticks as a natural reservoir are important vectors for ASFV transmission [2,3]. Since ASF was initially reported in Kenya in 1921, it has spread from Africa to the Americas, Europe, Asia and the Pacific [4]. ASF has become a serious threat for global swine industry and biodiversity [1,5]. However, the control of ASF continues to face numerous challenges due to the complexity of ASFV and the absence of effective ASF vaccines.

ASFV is the only known DNA arbovirus belonging to the family *Asfarviridae* and possesses a large genome of approximately 170–190 kb that encodes more than 150 viral proteins involved in viral entry, immune evasion and progeny virus production throughout the viral life cycle [2]. Due to random deletions and insertions of the variable region, the genomes of ASFV strains exhibit significant genetic diversity, even among different passages of ASFV from the same source [3]. ASFV isolates are classified into 24 genotypes based on the C-terminal sequence variation of the B646L gene, of which only genotypes I and II have been circulating outside Africa since 1961 [6,7,8]. Most notably, genotype II has emerged as the predominant circulating strain, resulting in severe socio-economic losses of the swine industry [9,10].

To control ASFV outbreaks, two commercial live-attenuated ASFV vaccines, AVAC ASF LIVE^®^ (AVAC, ASFV-G-ΔMGF, Van Lam, Vietnam) and NAVET-ASFVAC^®^ (Navetco, ASFV-G-ΔI177L, Ho Chi Minh City, Vietnam), were approved by Vietnam in 2022 [11]. However, the commercial vaccines provide poor cross-protection against various ASFV genotypes, particularly the circulating genotype I/II recombinant strains, and they also pose biosafety concerns related to potential virulence reversal or severe adverse effects in pregnant sows, fetuses and piglets [12,13]. Among different vaccine platforms, ASFV subunit vaccines based on CD2v, p72, p30 and p54 antigens have attracted widespread attention due to their neutralizing activity and safety [14,15]. But they still fail to provide sufficient protection in challenge experiments, even in the presence of neutralizing antibodies, which implies a need for a deeper understanding of ASFV proteins [16,17]. Furthermore, multiepitope recombinant antigens may be of great importance for the development of ASFV subunit vaccines.

In this study, unlike the previous epitope-mapping study utilizing mammalian cell-expressed ASFV pE146L [18], pE146L protein was expressed using the prokaryotic expression system to generate a panel of novel monoclonal antibodies through hybridoma fusion and cell subcloning screening. The linear epitopes of pE146L protein were finely mapped through progressive truncation analysis. These findings further complement the antigenic mapping of the pE146L protein and provide foundational data that may offer valuable insights for ASFV subunit vaccines and diagnostic reagents.

## 2. Materials and Methods

### 2.1. Genes, Cells and Sera

Based on ASFV China/2018/AnhuiXCGQ strain (GenBank: MK128995.1), the codon-optimized sequence of *pE146L* gene was synthesized by Sangon Biotech (Shanghai, China). Rosetta (DE3) competent cells were prepared in-house and used for protein expression. SP2/0 and 293T cells were cultured in RPMI 1640 and DMEM media (Gibco, Waltham, MA, USA), respectively. Both media were supplemented with 10% (*v*/*v*) FBS (VivaCell, Shanghai, China) and 1% (*v*/*v*) penicillin-streptomycin (Beyotime, Shanghai, China). The standard ASFV-positive swine serum was purchased from the China Institute of Veterinary Drug Control (Beijing, China). Additionally, an ASFV-negative and five ASFV-positive swine serum samples collected after 2018 were archived in our laboratory. All serum samples were handled and processed in strict accordance with the standard operating procedures for ASFV as mandated by the World Organisation for Animal Health (WOAH).

### 2.2. Expression and Purification of the Recombinant ASFV pE146L

The coding sequence of pE146L was amplified from the previously constructed pCGS3-E146L plasmid and inserted into the *EcoR*I/*Xho*I sites of the pET-32a. The resultant pET-32a-E146L plasmid was transformed into Rosetta (DE3) competent cells. For recombinant protein expression, the culture was induced with 0.2 mM IPTG and incubated at 16 °C for 16 h. Subsequently, the bacteria pellet was resuspended in Tris-HCl buffer (20 mM Tris-HCl pH 8.0, 150 mM NaCl) and lysed by sonication on ice. Following centrifugation, the clarified supernatant was collected and bound to Ni Sepharose excel affinity chromatography (Cytiva, Marlborough, MA, USA) for purification of the His-tagged pE146L. The production and purification of pE146L protein were verified by SDS-PAGE and Western blot using a mouse-derived anti-His mAb and standard ASFV-positive swine serum.

### 2.3. Generation of Monoclonal Antibodies Against pE146L

BALB/c female mice aged 6–8 weeks were provided by the Animal Experimental Center of Zhengzhou University and were housed under conventional conditions with a controlled temperature 25 ± 2 °C and 50 ± 10% humidity, with free access to standard diet and water throughout the study. Both mice were initially immunized subcutaneously (s.c.) with pE146L (20 μg/mouse) emulsified in Freund’s complete adjuvant (Sigma-Aldrich, St. Louis, MO, USA). Two booster immunizations were subsequently administered s.c. at 2-week intervals, using an identical protein amount formulated with Freund’s incomplete adjuvant (Sigma-Aldrich). One week after the final booster, tail vein blood samples were collected for the determination of serum antibody titers by indirect ELISA. Three days before cell fusion, the selected mice were intraperitoneally (i.p.) injected with pE146L without adjuvant. Splenocytes were then isolated and fused with SP2/0 cells using PEG-1500 (Cat no. 10783641001, Sigma-Aldrich). After three rounds of screening and subcloning, the positive hybridomas stably secreting monoclonal antibodies were identified by pE146L-based indirect ELISA.

### 2.4. Truncation and Expression of Short Peptides

To map the epitopes recognized by the obtained monoclonal antibodies, a stepwise truncation and expression strategy was employed for pE146L. In the initial truncation, the full-length pE146L was divided into three overlapping fragments with each adjacent fragment sharing a 15 amino acid overlap. The corresponding coding sequences were cloned into pGEX-6P-1 and then transformed into Rosetta (DE3) cells for expression as GST-tagged fusion proteins. The expression conditions were identical to those used for the full-length pE146L protein. Furthermore, the expression of these recombinant fusions was validated by immunoblotting using an anti-GST antibody.

Following initial truncation, fragments P1–P3 were further subdivided into overlapping peptides of 15–17 amino acids with 8–10-amino-acid overlaps. Complementary oligonucleotides pairs corresponding to each truncated peptide were synthesized, annealed, and cloned into the pEGFP-C1 vector. These resultant plasmids were transfected into 293T cells using PEI (Cat no. 40816ES02, Yeasen, Shanghai, China). The truncated peptides were expressed as EGFP-tagged fusions, which were confirmed by fluorescence microscopy (Nikon Ti2-U, Tokyo, Japan).

### 2.5. SDS-PAGE and Western Blot

Both the purified full-length pE146L and the soluble lysates of truncated fusion proteins obtained after sonication were resolved by 5–12% SDS-PAGE gel, transferred onto PVDF membranes (Millipore, Burlington, MA, USA), and blocked with 5% skim milk overnight at 4 °C. For immunoblotting, the full-length pE146L was incubated with an anti-His mAb (1:5000, Solarbio, Beijing, China) or standard ASFV-positive pig serum (1:2000), whereas the truncated fusion proteins were incubated with an anti-GST mAb (1:5000, Solarbio). All primary antibody incubations were performed for 1 h at room temperature (RT). After washing, the membranes were incubated with HRP-conjugated goat anti-mouse or goat anti-swine IgG (1:5000, Abbkine, Wuhan, China) for 1 h at RT. Protein bands were visualized using an eECL Western Blot Kit (Cat no. CW0049PM, CWBIO, Taizhou, China). Additionally, the purity of the full-length pE146L protein was assessed by Coomassie Brilliant Blue staining.

### 2.6. Indirect ELISA

Indirect ELISA was conducted to evaluate the reactivity of these mAbs toward the full-length pE146L and its truncated fragments. 96-wells microplates were coated overnight at 4 °C with different antigens diluted in CBS buffer (0.05 M, pH9.6, 100 μL/well). The coating antigens included purified full-length pE146L protein (2 μg/mL), soluble lysates of truncated fusion proteins (P1–P3, 1:10 dilution), BSA-conjugated synthetic peptides, as well as purified Trx-tagged (pET-32a) or GST-tagged (pGEX-6P-1) influenza virus proteins (2 μg/mL) used as negative controls. After blocking and washing, the microplates were incubated with hybridoma supernatants (serial dilutions for titer determination or 1:10 for epitope mapping) for 1 h at 37 °C. For serological assays, five ASFV-infected pig sera and one ASFV-negative pig serum (all diluted 1:100) were used as primary antibodies to evaluate the reactivity of BSA-conjugated synthetic peptides. After washing, the microplates were incubated with HRP-conjugated goat anti-mouse or goat anti-swine IgG (1:5000, Abbkine) for 1 h at 37 °C, depending on the species of the primary antibody. The reaction was developed using TMB substrate and terminated with 2 M H_2_SO_4_. The absorbance at 450 nm was measured using a SpectraMax iD5 microplate reader (Molecular Devices, San Jose, CA, USA). Antibody subclasses were identified with a commercial isotyping ELISA kit (Cat no. PK20003, Proteintech, Wuhan, China) following the manufacturer’s instructions.

### 2.7. Immunofluorescence Assay

293T cells were transfected using PEI with recombinant plasmids expressing full-length pE146L (pcDNA3.1-pE146L-Myc-His), various truncated EGFP-tagged fusion proteins (P1-1 to P1-4, P2-1 to P2-7, and P3-1 to P3-4), or the empty vector control. At 48 h post-transfection, cells were fixed with 4% Paraformaldehyde (Cat no. P1110, Solarbio), permeabilized with Triton X-100 (Cat no. P0096-500 mL, Beyotime), and blocked with 5% skim milk overnight at 4 °C. For the 293T cells expressing full-length pE146L, hybridoma supernatants and pE146L-immunized mouse serum were applied as primary antibodies, followed by FITC-conjugated goat anti-mouse IgG (1:200, Abbkine) and DAPI nuclear staining. For the 293T cells expressing truncated EGFP-tagged fusion proteins, the corresponding hybridoma supernatants were applied as primary antibodies, followed by DyLight 594-conjugated goat anti-mouse IgG (1:200, Abbkine). All antibodies were incubated for 1 h at RT. Finally, the signals were observed under a Nikon Ti2-U fluorescence microscope (Tokyo, Japan).

### 2.8. Dot-Blot Assay

Dot-blot assays were performed to evaluate the reactivity of these mAbs toward truncated fusion proteins and synthetic peptides, and to evaluate the antigenicity of these identified epitopes using ASFV-infected pig sera. Briefly, soluble lysates of truncated fusion proteins (P1–P3), purified full-length pE146L (positive control), and purified Trx- or GST-tagged influenza virus protein (negative control) were spotted onto nitrocellulose (NC) membranes and air-dried. Synthetic peptides were also directly spotted onto NC membranes. After blocking with 5% skim milk for 2 h at 37 °C, the membranes were incubated overnight at 4 °C with hybridoma supernatants to assess the reactivity of the generated mAbs against the truncated proteins and synthetic peptides. For serological antigenicity assays, these synthetic peptides were incubated with five ASFV-infected pig sera (1:100), with one ASFV-negative pig serum (1:100) serving as a negative control. Following washing with PBST, the membranes were incubated with HRP-conjugated goat anti-mouse or goat anti-swine IgG (1:5000, Abbkine) for 1 h at RT. Immunoreactive signals were visualized using an eECL Western Blot Kit.

### 2.9. Biological Information Analysis

The amino acid sequences of pE146L were aligned using CLUSTALW (https://www.genome.jp/tools-bin/clustalw, accessed on 20 October 2025), and the alignment results were analyzed and graphically represented with Jalview (https://www.jalview.org/jalview-js/jalviewjs/, accessed on 20 October 2025) to assess the conservation of the identified epitopes among ASFV strains. In addition, the three-dimensional structure of the full-length pE146L protein was predicted using AlphaFold 3 (https://deepmind.google/science/alphafold/, accessed on 20 October 2025), and spatial distribution of the identified epitopes on the pE146L structure was visualized using PyMOL software (version 3.0.3, Schrödinger, LLC., New York, NY, USA).

## 3. Results

### 3.1. Expression and Immunogenicity of Recombinant pE146L

**Figure 1:**
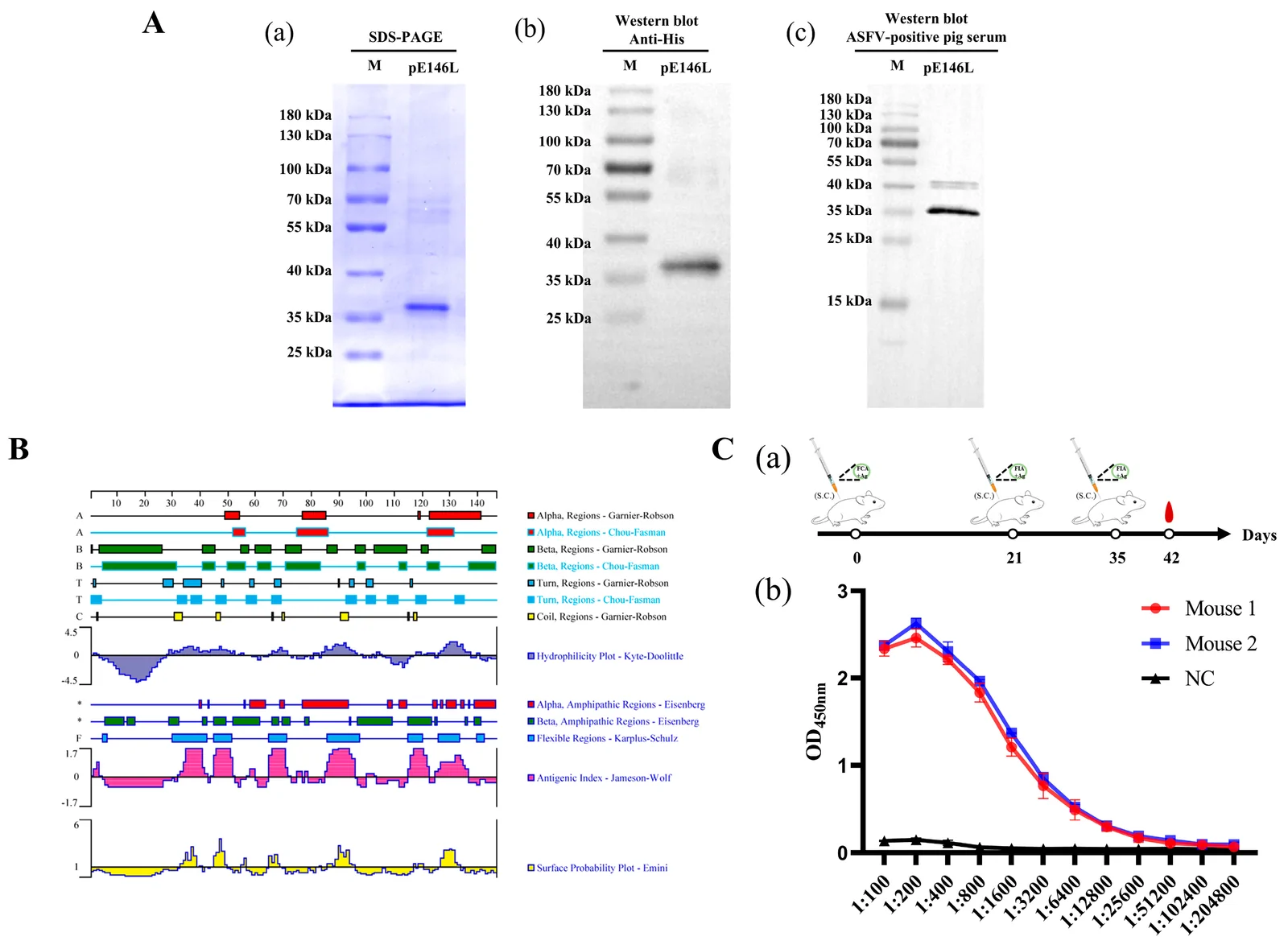
Expression and immunogenicity of the ASFV pE146L protein. (**A**) The purified pE146L was analyzed by SDS-PAGE (**a**) and verified by Western blotting using anti-His antibody (**b**) and standard ASFV-positive pig serum (1:2000) (**c**). M, Marker. (**B**) Secondary structure and Antigenicity analysis of the pE146L by Protean. * indicate the decision thresholds for Eisenberg amphipathic region predictions, where colored blocks mark regions with significant amphipathicity. (**C**) The immunogenicity of the pE146L. (**a**) The immunization schedule of the BALB/c mice immunized with the purified pE146L emulsified in Freund’s adjuvant. (**b**) The pE146L-specific antibody titers of the immunized mice were determined by indirect ELISA. NC, the pre-immune mouse.

In this study, the constructed pET-32a-E146L plasmid was transformed into Rosetta (DE3) bacteria for protein expression. The expressed His-tagged pE146L protein was purified using Ni-Affinity Chromatography Column, and the purified pE146L was analyzed by Coomassie blue-stained SDS-PAGE and Western blot. As shown in Figure 1A(a), the purified pE146L protein migrated as a single band with an apparent molecular weight of approximately 36 kDa, with no detectable impurity bands, and Western blot analysis confirmed that the recombinant protein was specifically recognized by anti-His mAb (Figure 1A(b)) and standard ASFV-positive pig serum (Figure 1A(c)). Although a faint band at approximately 40 kDa was observed in the reaction with the ASFV-positive pig serum, which is potentially attributable to a cross-reaction between the antiserum and trace residual host proteins. Furthermore, Figure 1B also shows that the extracellular domain of pE146L exhibited good antigenicity. This is supported by the significant correlation and overlap between the regions of high Antigenic Index (Jameson-Wolf), high Surface Probability (Emini), and favorable Hydrophilicity and Flexibility across this region. Subsequently, the immunogenicity of the purified pE146L protein was evaluated in female BALB/c mice (Figure 1C(a)), and indirect ELISA analysis of serum samples collected one week after the third immunization showed that both immunized mice developed higher pE146L-specific antibody titers of 1:12800, which were markedly higher than those of the pre-immune mouse (Figure 1C(b)). These results indicated that the recombinant protein had good immunogenicity and can serve as an immunogen for pE146L-specific monoclonal antibodies production.

### 3.2. Screening and Identification of mAbs Against pE146L

**Figure 2:**
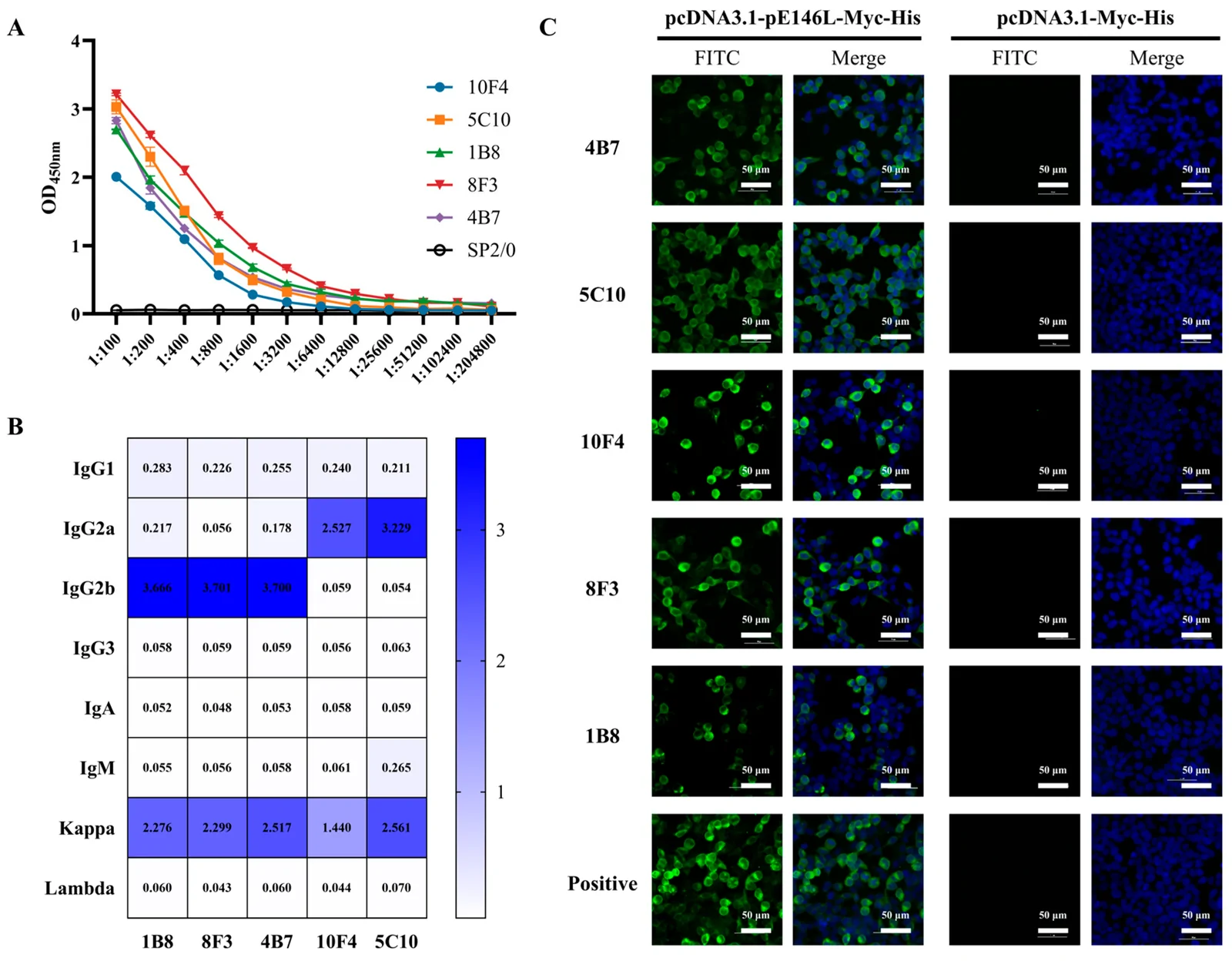
Characterization and identification of the pE146L-specific monoclonal antibodies (mAbs). (**A**) The antibody titers of five mAbs (10F4, 5C10, 1B8, 8F3 and 4B7) were determined by indirect ELISA. The hybridoma cell supernatants were utilized as the primary antibodies, while the SP2/0 cell supernatant served as the negative control. (**B**) The subtypes of all five mAbs were determined using the Mouse Monoclonal Antibody Isotyping Kit (Cat no. PK20003, Proteintech), according to the manufacturer’s instructions. (**C**) The specificity of pE146L-specific mAbs was verified by IFA. 293T cells were transfected with pcDNA3.1-pE146L-Myc-His or pcDNA3.1-Myc-His plasmids. Cells were fixed and permeabilized at 48 h post-transfection. Next, the cells were sequentially incubated with either the hybridoma cell supernatants or serum from the immunized mice (as primary antibodies), followed by FITC-conjugated goat anti-mouse IgG (as the secondary antibody) for 1 h each at room temperature. Cell nuclei were stained with DAPI (1 μg/mL) for 2 min at room temperature. (scale bar = 50 μm).

Monoclonal antibodies against pE146L were generated by fusing splenocytes from the immunized mice with SP2/0 cells. After three rounds of screening and subcloning, five hybridoma clones designated 10F4, 5C10, 1B8, 8F3, and 4B7 were identified using a pE146L-based indirect ELISA. The antibody titers of five mAbs ranged from 1:6400 to 1:25,600 (Figure 2A). Antibody isotyping revealed that three mAbs (1B8, 8F3, and 4B7) belonged to the IgG2b subclass with Kappa light chains, two mAbs (10F4 and 5C10) belonged to the IgG2a subclass with Kappa light chains (Figure 2B). Furthermore, the binding specificity of these mAbs was confirmed by IFA assay. As demonstrated in Figure 2C, strong FITC fluorescence signals were detected in 293T cells transfected with pcDNA3.1-pE146L-Myc-His, while the signals were absent in 293T cells transfected with empty vectors. The specific reactivity indicated that all five mAbs specifically recognized the pE146L protein.

### 3.3. Preliminary Epitopes Mapping of the pE146L Protein

**Figure 3:**
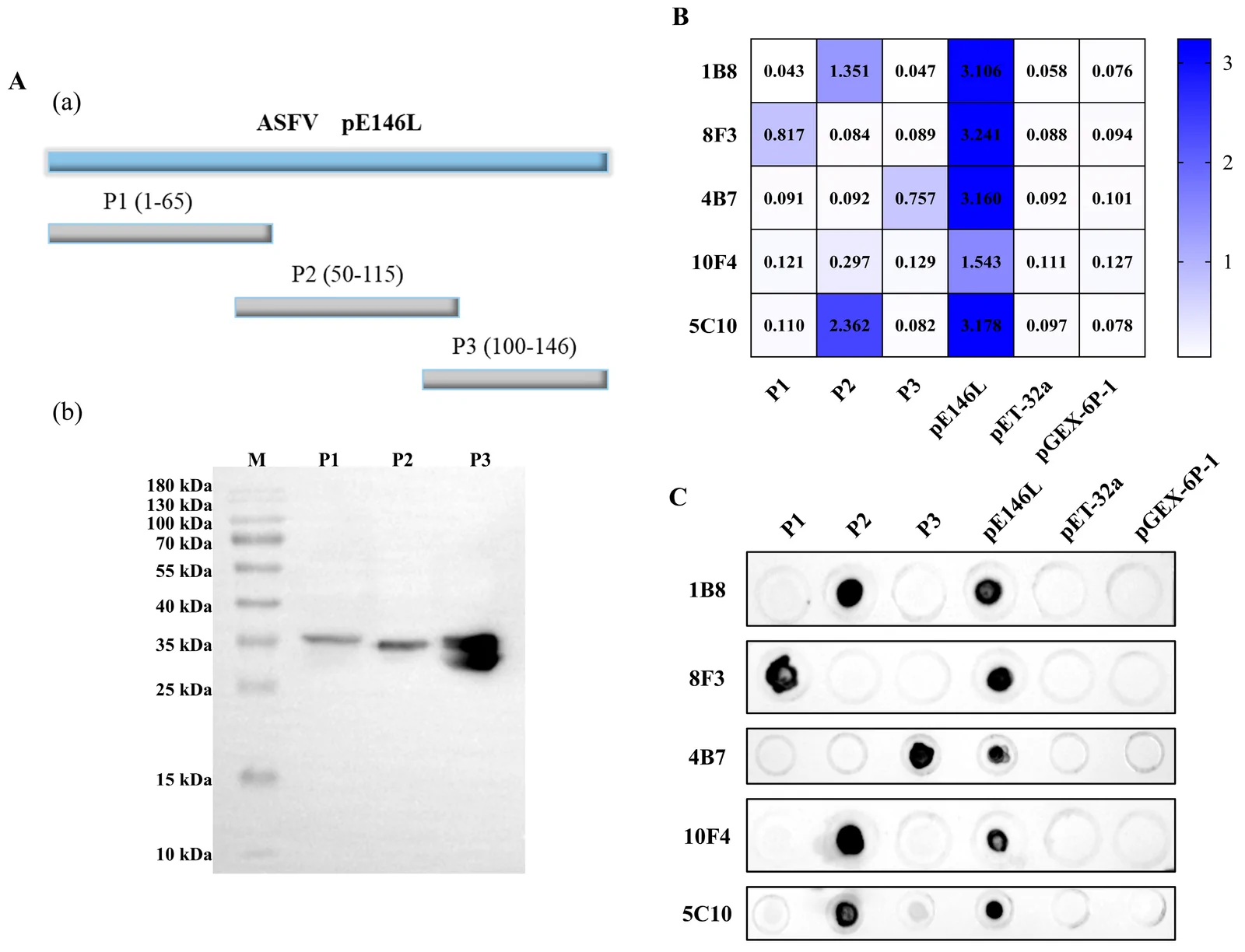
Preliminary mapping of the antigenic epitopes recognized by all five pE146L-specific mAbs. (**A**) Truncation and expression of the pE146L protein. (**a**) Schematic of the initial truncation strategy for the pE146L. (**b**) Western blot verification of the three truncated GST-fusion proteins expressed in Rosetta (DE3) using an anti-GST antibody. Lane M, Marker. Lanes P1–P3, three truncated fusion proteins. (**B**) ELISA analysis of the reactivity of the five mAbs against the three truncated fusion proteins. (**C**) Dot-blot analysis of the reactivity of the five mAbs against the three truncated fusion proteins. pE146L, positive control. pET-32a and pGEX-6P-1, negative controls.

To map the linear epitopes of pE146L, the full-length protein was first divided into three fragments (P1–P3), as illustrated in Figure 3A(a). The truncated fragments were expressed as GST-tagged fusion proteins in Rosetta (DE3) bacteria, and Western blot analysis confirmed the successful expression of these three fusion proteins (Figure 3A(b)). Indirect ELISA and Dot-blot assays (Figure 3B,C) revealed that mAbs 5C10, 1B8, and 10F4 recognized the P2, with 10F4 showing weak reactivity, mAb 8F3 recognized the P1, whereas mAb 4B7 recognized the P3. All mAbs reacted with the full-length pE146L protein but not with tag proteins expressed from the pET-32a or pGEX-6p-1 vectors. Taken together, these results indicated that the epitopes recognized by the five mAbs were distributed across N-, central-, and C-terminal regions of the pE146L protein.

### 3.4. Identification of the Fine Epitopes Recognized by All Five mAbs

**Figure 4:**
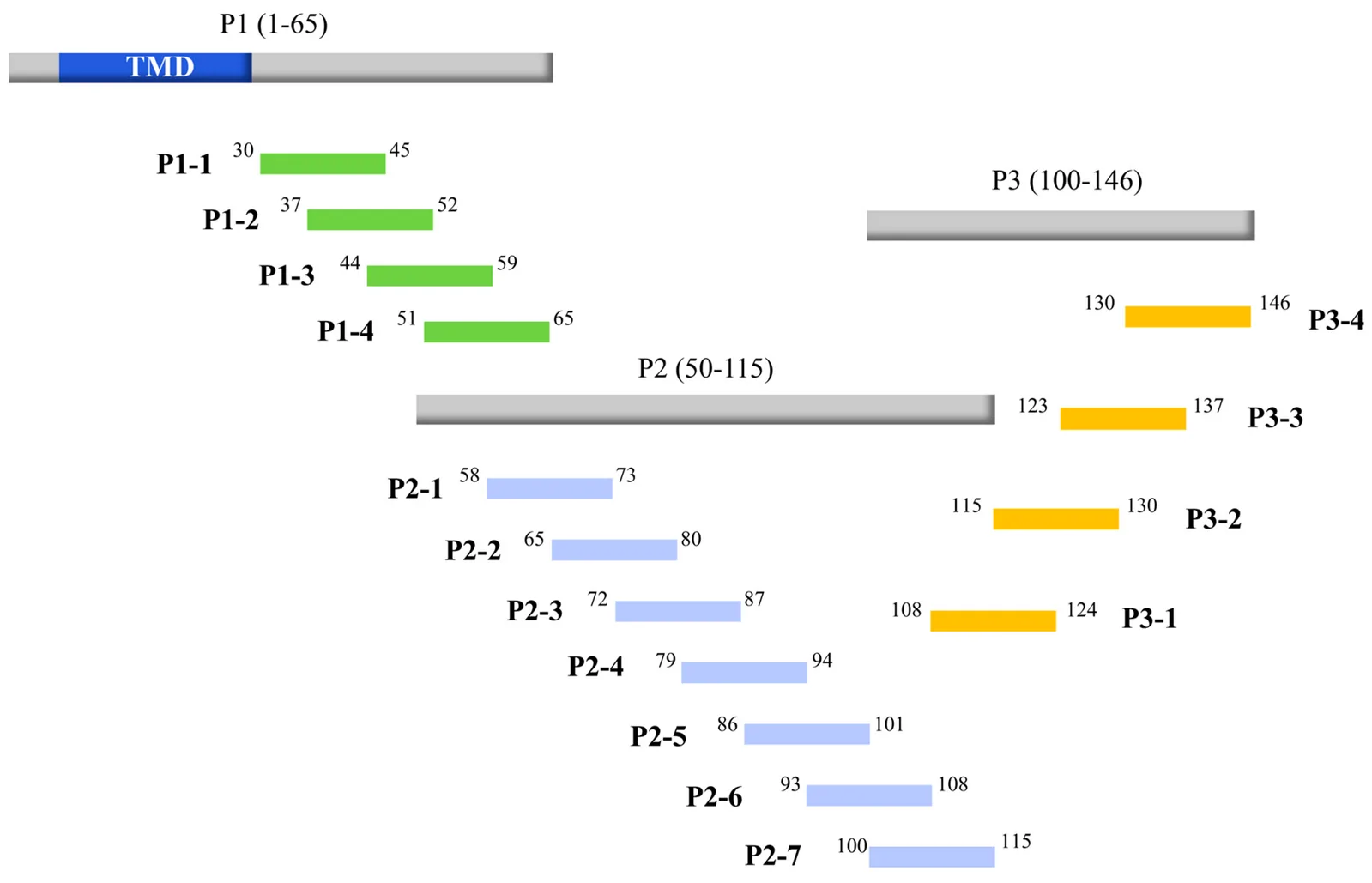
Design of secondary truncations of pE146L for fine mapping of antigenic epitopes.

**Figure 5:**
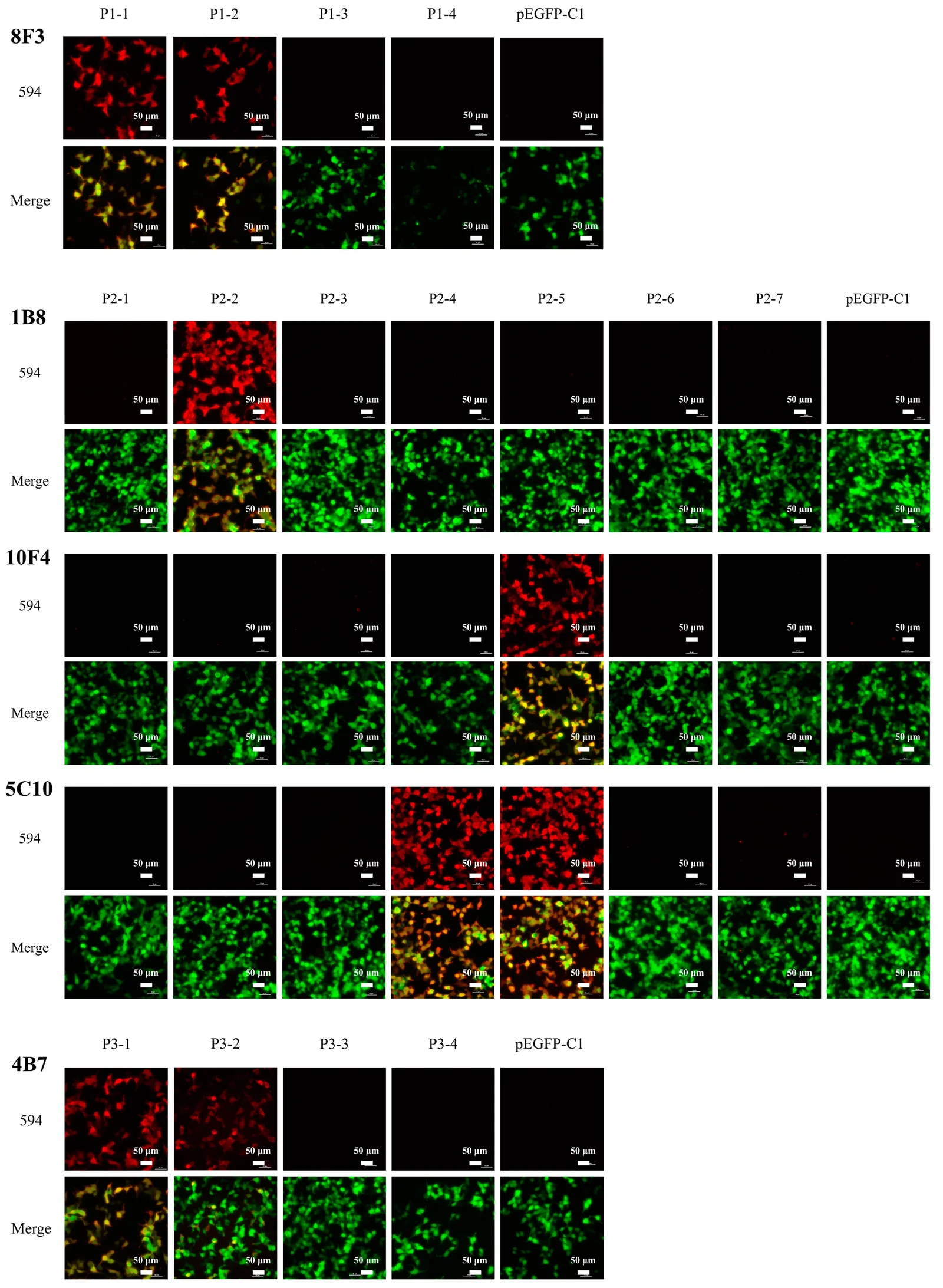
Reactivity of five monoclonal antibodies with their respective further truncated overlapping peptides of the pE146L protein was determined by indirect immunofluorescence assay (IFA). 293T cells were transfected with the recombinant plasmids expressing further truncated overlapping peptides (P1-1 to P1-4, P2-1 to P2-7, and P3-1 to P3-4) or with pEGFP-C1 vector as a control. At 48 h post-transfection, cells were fixed, permeabilized, and probed with the respective hybridoma supernatants, followed by incubation with DyLight 594-conjugated goat anti-mouse IgG (1:200). Green fluorescence (EGFP) indicated successful expression of EGFP-peptide fusion proteins, whereas red fluorescence (594) indicated specific peptide-antibody binding. The absence of red signal in the pEGFP-C1 control group confirmed antibody specificity. Five monoclonal antibodies: 8F3, 1B8, 10F4, 5C10, and 4B7. (scale bar = 50 μm).

Based on the results of the primary truncation analysis, the P1, P2, and P3 fragments were subsequently subdivided into further truncated overlapping peptides each for fine epitope mapping (Figure 4). The shorter peptides were expressed as EGFP-tagged fusion proteins and analyzed by IFA. As demonstrated in Figure 5, clear green fluorescence signals were detected in 293T cells for all EGFP-fusion constructs except P1-4, confirming that these truncated overlapping peptides were effectively expressed at the C-terminus of the EGFP tag. In addition, subsequent IFA assays revealed the specific reactivity of the five monoclonal antibodies (mAbs) with these truncated overlapping peptides. Specifically, mAb 8F3 recognized both P1-1 and P1-2, mAb 1B8 recognized P2-2, mAb 10F4 recognized P2-5, and mAb 5C10 reacted both P2-4 and P2-5. Meanwhile, mAb 4B7 exhibited reactivity with the P3-1 and P3-2, though with noticeably weaker intensity observed for P3-2. Collectively, these findings indicated that the linear epitopes recognized by these five mAbs (8F3, 1B8, 10F4, 5C10, and 4B7) were finely mapped to amino acids 37–45, 65–80, 86–101, 86–94, and 115–124 of the pE146L protein, respectively.

### 3.5. Reactivity of Synthetic Peptides Toward mAbs and ASFV-Infected Positive Pig Sera

**Figure 6:**
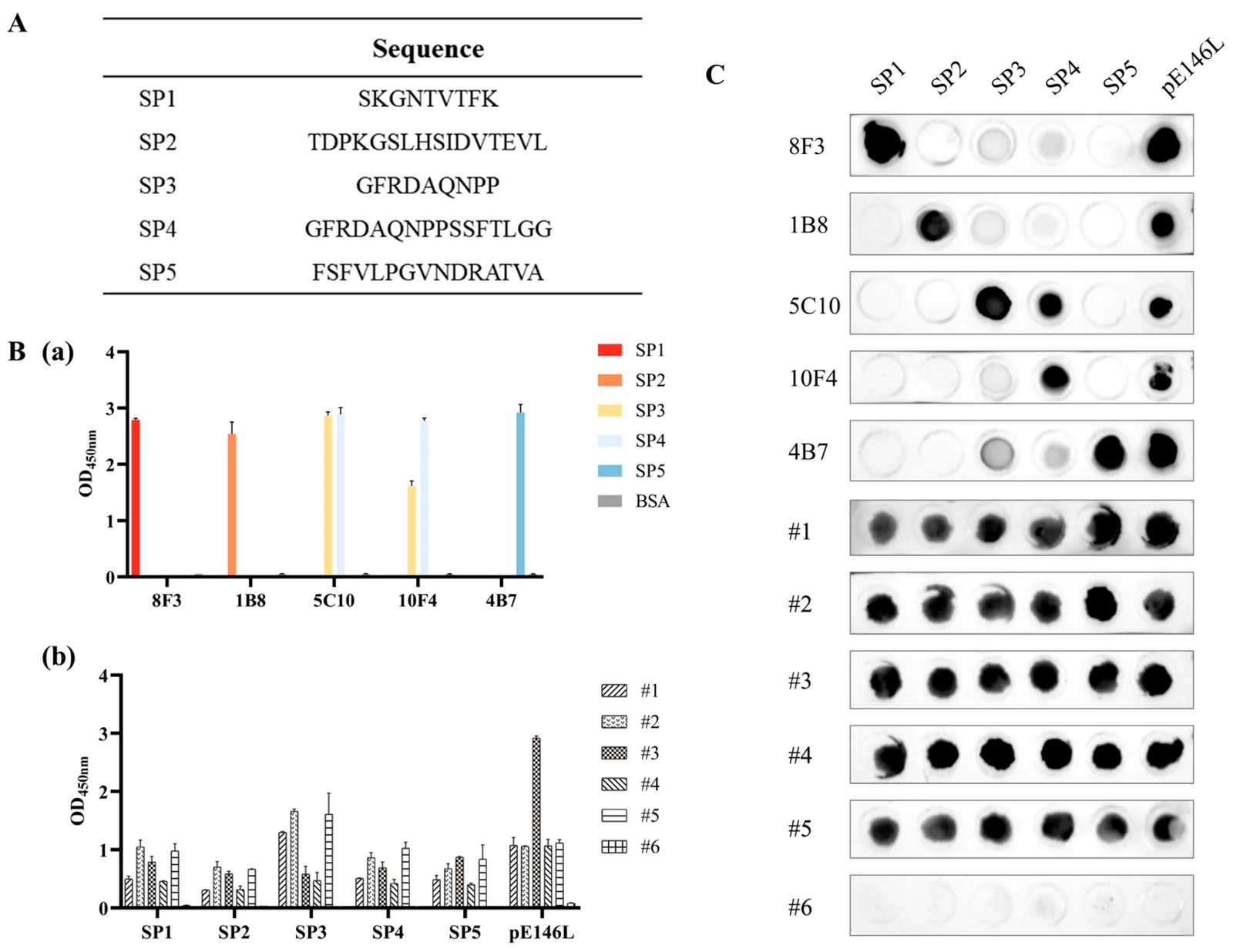
Reactivity of the synthetic peptides toward monoclonal antibodies and pig sera. (**A**) Amino acid sequences of synthetic peptides SP1–SP5. (**B**) Antigenicity of the synthetic peptides toward monoclonal antibodies (**a**) and pig sera (**b**) in ELISA. Synthetic peptides SP1–SP5 were conjugated to BSA carrier protein using glutaraldehyde, and then coated onto microplates at 1:50 dilution. Hybridoma supernatant (1:10) and pig sera (1:100) were used as primary antibodies. BSA served as the negative control, and purified pE146L protein served as the positive control. Note: The OD_450nm_ values for the BSA control in (**a**) were close to zero, and the data for pig sera in (**b**) are presented after subtracting the corresponding BSA background values. (**C**) Antigenicity of the synthetic peptides toward monoclonal antibodies and pig sera in Dot-blot analysis. Synthetic peptides SP1–SP5 were spotted onto NC membranes and probed with hybridoma supernatants or pig serum (1:100), followed by HRP-conjugated secondary antibodies. Signals were visualized by an eECL. 8F3, 1B8, 5C10, 10F4, and 4B7: monoclonal antibodies. #1–#5: ASFV-infected positive pig sera, #6: negative pig serum.

Based on the secondary truncation analysis, the amino acid sequences of the mapped epitopes were synthesized by GL Biochem (Shanghai) Ltd. (Shanghai, China) (Figure 6A). Notably, the synthesized sequence of SP5 was designated as residues 110–124 instead of the 115–124 overlap, which was because mAb 4B7 exhibited stronger reactivity with fragment P3-1 than with P3-2 in Figure 5, indicating that the N-terminal flanking residues (aa 110–114) may enhance antibody binding or peptide presentation. Subsequently, the reactivity of synthetic peptides SP1–SP5 toward monoclonal antibodies and pig sera were evaluated by ELISA (Figure 6B) and Dot-blot assays (Figure 6C). For monoclonal antibodies, Figure 6B(a) shows that the BSA-conjugated synthetic peptides SP1–SP5 were specifically recognized by mAbs 8F3, 1B8, 5C10, 10F4, and 4B7, respectively. Notably, because SP4 contains the entire SP3 sequence, mAb 5C10 recognized both BSA-conjugated synthetic peptides SP3 and SP4, whereas mAb 10F4 showed stronger reactivity with SP4 than with SP3, confirming that SP4 represents the minimal epitope recognized by 10F4.

For pig sera, all ASFV-infected positive pig sera (#1–#5) showed strong reactivity with the full-length pE146L protein and detectable reactivity with all five BSA-conjugated synthetic peptides (SP1–SP5). Among them, sera #1, #2, and #5 exhibited particularly strong reactivity with SP3, sera #2, #3, and #5 also showed moderate but comparable reactivity with SP1 and SP4. As expected, serum #6, a negative control, showed no reactivity with the full-length pE146L protein. Consistent results were observed in the Dot-blot assay using unconjugated synthetic peptides (Figure 6C). Collectively, these results confirmed the immunogenicity of synthetic peptides SP1–SP5, with SP3, SP1, and SP4 showing strong antigenicity.

### 3.6. Bioinformatics Analysis of the Identified pE146L Epitopes

**Figure 7:**
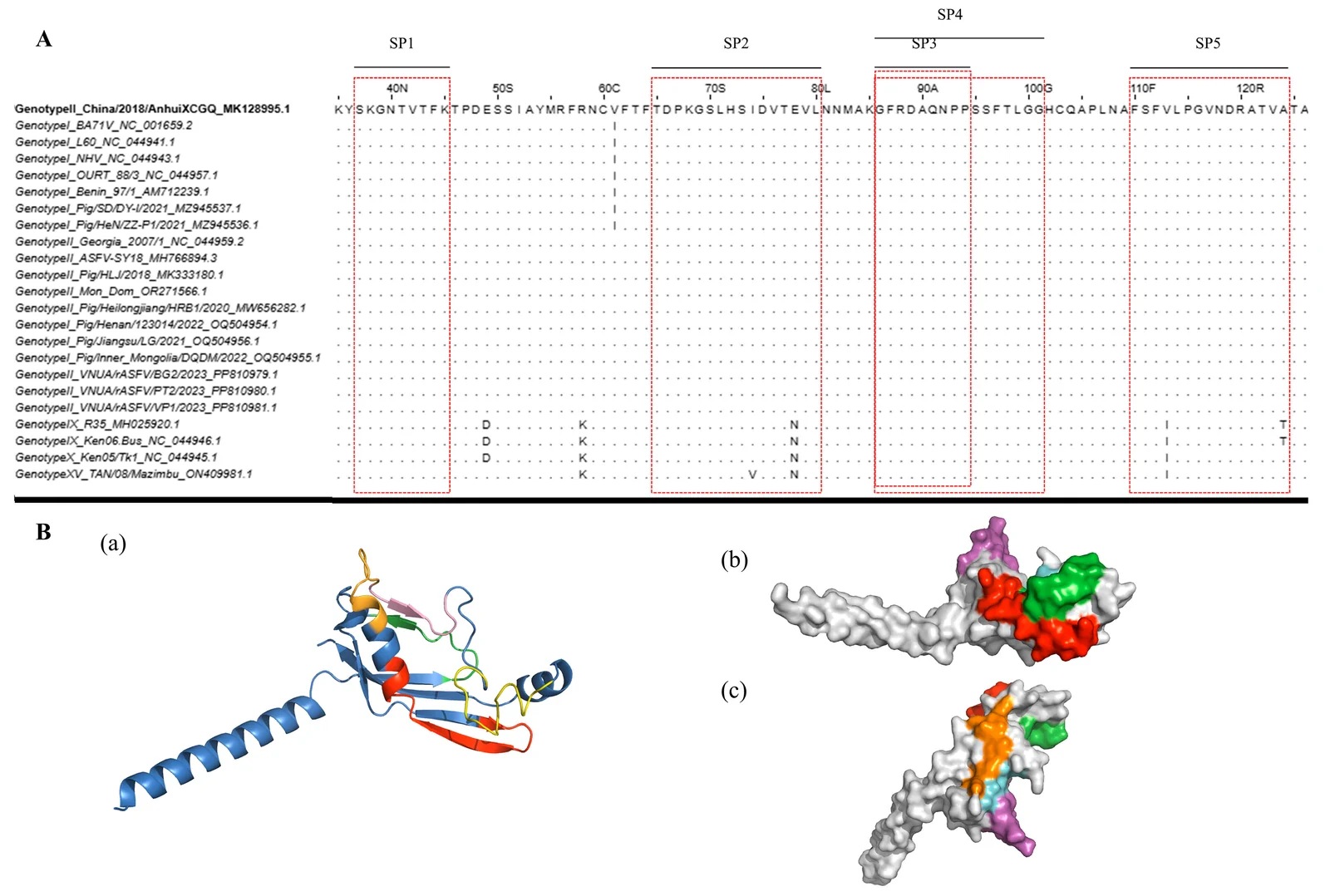
Conservation analysis and visualization of B-cell epitopes on the pE146L protein. (**A**) Multiple sequence alignment of pE146L proteins from different ASFV strains was performed using CLUSTALW and visualized with Jalview, with synthetic peptides SP1–SP5 highlighted in red boxes. (**B**) Spatial localization of identified B-cell epitopes on the AlphaFold 3-predicted pE146L structure. The pE146L structure was visualized in PyMOL as cartoon (**a**) and surface (**b**,**c**) models. SP1–SP5 were colored orange, red, magenta, magenta with cyan extension (SP4 included SP3), and green, respectively.

To evaluate the conservation of the identified B-cell epitopes among different ASFV strains, a total of twenty-two pE146L sequences representing genotypes I, II, IX, X, XV, and I/II recombinant strains were retrieved from the NCBI database and aligned using CLUSTALW with default parameters. The alignment results showed that epitopes SP1, SP3, and SP4 were highly conserved among all genotypes, whereas SP2 and SP5 were conserved mainly in genotypes I and II (Figure 7A). Furthermore, structural mapping demonstrated that all identified epitopes were distributed on the outer surface of the pE146L protein (Figure 7B), suggesting good surface accessibility for antibody recognition. However, a small portion of SP4 appears to be partially buried, which may reduce its spatial accessibility compared with SP3. This structural characteristic may provide one possible explanation for the relatively weak reactivity shown in Figure 3B, where mAb 10F4 exhibited limited binding to the initial P2 fragment during the early stage of epitope mapping. Nevertheless, as pE146L functions as an inner envelope protein within the complex architecture of the ASFV virion, these computational accessibility claims remain predictive. The actual exposure and antibody accessibility of these epitopes on native virions require further experimental confirmation.

## 4. Discussion

African swine fever (ASF) remains one of the most devastating viral diseases threatening the global swine industry. Since the first outbreak in China in 2018, ASF has caused tremendous economic losses to the country’s pig industry [19,20]. The absence of safe and effective vaccines remains the major obstacle to ASF control. Furthermore, genotypes I and I/II recombinant ASFV strains were first isolated from pig farms in China in 2021 and 2023, respectively [21,22], indicating that the epidemic pattern of ASF has shifted from the dominance of genotype II to the co-circulation of genotypes I and II [23,24]. The genetic diversification increases the complexity of epidemiological surveillance and poses new challenges for disease prevention and control [25]. In this context, the design of multiepitope antigens represents a promising strategy for the development of subunit vaccines and serological diagnostic assays [26]. Therefore, understanding the antigenic and structural characteristics of ASFV proteins is essential for the development of effective diagnostic tools and vaccines.

ASFV is a large double-stranded DNA virus characterized by a multilayered structure and an icosahedral morphology [27]. Extensive studies have been conducted to elucidate the structural and functional features of ASFV proteins [28,29,30,31]. Among them, pE146L is a late structural protein localized in the inner envelope of the ASFV virion. It plays an essential role in viral replication by promoting the formation of viral factories [32]. In a previous study, Zhang et al. generated three monoclonal antibodies (mAbs) by immunizing BALB/c mice with BHK-21 cell-expressed pE146L protein, and the fine epitopes recognized by these mAbs were mapped to amino acid residues 30–41, 48–61, and 138–146 of the pE146L protein. Furthermore, serological analysis of ASFV-infected pig sera revealed that the epitope (^30^GWSPFKYSKGNT^41^) represents an immunodominant B-cell epitope [18]. However, relying on a single antigenic site can lead to false-negative results due to individual variations in herd immune responses or potential sequence mutations across shifting ASFV field strains. Therefore, further comprehensive research on the pE146L protein is critically needed to discover additional functional regions.

In this study, five novel monoclonal antibodies (mAbs, 4B7, 5C10, 10F4, 1B8, and 8F3) were obtained by immunizing BALB/c mice with *E.coli*-expressed pE146L protein. Using truncated overlapping fragments, the fine epitopes recognized by mAbs 8F3, 1B8, 5C10, 10F4, and 4B7 were mapped to amino acid residues 37–45 (SP1), 65–80 (SP2), 86–94 (SP3), 86–101 (SP4), and 115–124 of the pE146L protein, respectively. Notably, the epitope SP1 overlaps with the immunodominant epitope previously reported by Zhang et al. [18]. This consistency validates the reliability of our mapping strategy and confirms the antigenic importance of this region. In addition, four novel epitopes were identified and represented by synthetic peptides SP2 to SP5, among which SP5 was designed as an extended peptide spanning aa110–124, incorporating the N-terminal extension (aa110–114) based on initial fragment reactivity, as it was hypothesized to potentially facilitate antibody binding or peptide presentation. These synthetic peptides reacted strongly with both their respective mAbs and ASFV-positive swine sera, providing new evidence that pE146L harbors multiple antigenic sites. Regrettably, the specific critical amino acid residues essential for antibody binding within these mapped regions have not yet been individually characterized via alanine scanning mutagenesis, which remains a limitation of the current study that warrants further fine-mapping refinement.

Furthermore, sequence alignment further demonstrated that these epitopes are highly conserved among the predominant genotypes I and II ASFV strains as well as the recently emerged genotype I/II recombinant strains, suggesting their theoretical potential as broadly reactive antigenic targets. However, the conservation of these mapped epitopes remains to be rigorously validated in field diagnostic applications. Although six swine serum samples were utilized in this study to confirm the preliminary antigenicity of these epitopes, this limited sample size and the restricted background profiles are far from sufficient to thoroughly evaluate their real-world diagnostic performance. Therefore, verifying these individual epitopes against a broader collection of field-positive sera is critically necessary to better assess their diagnostic applicability. On this basis, systematic comparative evaluations including both our newly identified and previously reported epitopes are further warranted to determine their individual or synergistic performance, which will facilitate the screening of the most effective epitope or epitope combinations for practical field applications. Overall, the successful mapping of multiple distinct antigenic sites on the pE146L protein in this study provides a foundational framework for understanding ASFV antigenicity and offers preliminary multi-target candidates that warrant further validation in larger diagnostic cohorts.

## 5. Conclusions

In summary, we generated five pE146L-specific monoclonal antibodies and finely mapped their linear epitopes (aa 37–45, 65–80, 86–94, 86–101, and 115–124) using a panel of truncated overlapping peptides. Importantly, the synthetic peptides derived from these epitopes, including SP1–SP4 and the extended peptide SP5 spanning aa110–124, exhibited strong reactivity with ASFV-infected positive pig sera, demonstrating that these epitopes are antigenic and recognized during ASFV infection. These findings identify novel epitope candidates that may serve as potential components for future multi-epitope assays, pending further evaluation with larger, well-characterized serum panels.

## Data Availability

The original contributions presented in this study are included in the article. Further inquiries can be directed to the corresponding author.

## References

[1] Chathuranga K., Lee J.-S. (2023). African Swine Fever Virus (ASFV): Immunity and Vaccine Development. Vaccines.

[2] Lv T., Xie X., Song N., Zhang S., Ding Y., Liu K., Diao L., Chen X., Jiang S., Li T. (2022). Expounding the Role of Tick in Africa Swine Fever Virus Transmission and Seeking Effective Prevention Measures: A Review. Front. Immunol..

[3] Bao J., Zhang Y., Shi C., Wang Q., Wang S., Wu X., Cao S., Xu F., Wang Z. (2022). Genome-Wide Diversity Analysis of African Swine Fever Virus Based on a Curated Dataset. Animals.

[4] African Swine Fever. https://www.woah.org/en/disease/african-swine-fever/.

[5] Hakizimana J.N., Yona C., Makange M.R., Adamson E.K., Ntampaka P., Uwibambe E., Gasana M.N., Ndayisenga F., Nauwynck H., Misinzo G. (2025). Complete Genome Analysis of the African Swine Fever Virus Genotypes II and IX Responsible for the 2021 and 2023 Outbreaks in Rwanda. Front. Vet. Sci..

[6] O’Donnell V., Spinard E., Xu L., Berninger A., Barrette R.W., Gladue D.P., Faburay B. (2024). Full-Length ASFV B646L Gene Sequencing by Nanopore Offers a Simple and Rapid Approach for Identifying ASFV Genotypes. Viruses.

[7] Domelevo Entfellner J.-B., Okoth E.A., Onzere C.K., Upton C., Njau E.P., Höper D., Henson S.P., Oyola S.O., Bochere E., Machuka E.M. (2024). Complete Genome Sequencing and Comparative Phylogenomics of Nine African Swine Fever Virus (ASFV) Isolates of the Virulent East African P72 Genotype IX without Viral Sequence Enrichment. Viruses.

[8] Njau E.P., Domelevo Entfellner J.-B., Machuka E.M., Bochere E.N., Cleaveland S., Shirima G.M., Kusiluka L.J., Upton C., Bishop R.P., Pelle R. (2021). The First Genotype II African Swine Fever Virus Isolated in Africa Provides Insight into the Current Eurasian Pandemic. Sci. Rep..

[9] Ge S., Li J., Fan X., Liu F., Li L., Wang Q., Ren W., Bao J., Liu C., Wang H. (2018). Molecular Characterization of African Swine Fever Virus, China, 2018. Emerg. Infect. Dis..

[10] Kruszyński M., Śróda K., Juszkiewicz M., Siuda D., Olszewska M., Woźniakowski G. (2023). Nine Years of African Swine Fever in Poland. Viruses.

[11] Diep N.V., Duc N.V., Ngoc N.T., Dang V.X., Tiep T.N., Nguyen V.D., Than T.T., Maydaniuk D., Goonewardene K., Ambagala A. (2024). Genotype II Live-Attenuated ASFV Vaccine Strains Unable to Completely Protect Pigs Against the Emerging Recombinant ASFV Genotype I/II Strain in Vietnam. Vaccines.

[12] van den Born E., Olasz F., Mészáros I., Göltl E., Oláh B., Joshi J., van Kilsdonk E., Segers R., Zádori Z. (2025). African Swine Fever Virus Vaccine Strain Asfv-G-∆I177l Reverts to Virulence and Negatively Affects Reproductive Performance. npj Vaccines.

[13] Zhou X., Fan J., Guo X., Chen T., Yang J., Zhang Y., Mi L., Zhang F., Miao F., Li M. (2023). Comparison of Genotype II African Swine Fever Virus Strain SY18 Challenge Models. Viruses.

[14] Zhang Y., Mei X., Zhang C., Wang H., Xie X., Zhang Z., Feng Z. (2024). ASFV Subunit Vaccines: Strategies and Prospects for Future Development. Microb. Pathog..

[15] Lu Z., Zhong D., Song X., Lan J., Wang Y., Luo R., Chen S., Huang R., Qiu H.-J., Li Y. (2026). A Pool of Ferritin Nanoparticles Delivering Six Proteins of African Swine Fever Virus Induces Robust Humoral and Cellular Immune Responses in Pigs. Vaccines.

[16] Chandana M.S., Nair S.S., Chaturvedi V.K., Abhishek, Pal S., Charan M.S.S., Balaji S., Saini S., Vasavi K., Deepa P. (2024). Recent Progress and Major Gaps in the Vaccine Development for African Swine Fever. Braz. J. Microbiol..

[17] Wang L., Shi J. (2026). African Swine Fever: Vaccine Advancement and Major Gaps. Microorganisms.

[18] Zhang S.-J., Niu B., Liu S.-M., Bu Z.-G., Hua R.-H. (2024). Identification of Linear B Cell Epitopes on the E146L Protein of African Swine Fever Virus with Monoclonal Antibodies. Virol. J..

[19] Yan Z., Wang M., Li X., Jiang H. (2023). Impact of African Swine Fever Epidemic on the Cost Intensity of Pork Production in China. Agriculture.

[20] Zhao D., Liu R., Zhang X., Li F., Wang J., Zhang J., Liu X., Wang L., Zhang J., Wu X. (2019). Replication and Virulence in Pigs of the First African Swine Fever Virus Isolated in China. Emerg. Microbes Infect..

[21] Sun E., Huang L., Zhang X., Zhang J., Shen D., Zhang Z., Wang Z., Huo H., Wang W., Huangfu H. (2021). Genotype I African Swine Fever Viruses Emerged in Domestic Pigs in China and Caused Chronic Infection. Emerg. Microbes Infect..

[22] Zhao D., Sun E., Huang L., Ding L., Zhu Y., Zhang J., Shen D., Zhang X., Zhang Z., Ren T. (2023). Highly Lethal Genotype I and II Recombinant African Swine Fever Viruses Detected in Pigs. Nat. Commun..

[23] Xin G., Kuang Q., Le S., Wu W., Gao Q., Gao H., Xu Z., Zheng Z., Lu G., Gong L. (2023). Origin, Genomic Diversity and Evolution of African Swine Fever Virus in East Asia. Virus Evol..

[24] Le V.P., Nguyen V.T., Le T.B., Mai N.T.A., Nguyen V.D., Than T.T., Lai T.N.H., Cho K.H., Hong S.-K., Kim Y.H. (2024). Detection of Recombinant African Swine Fever Virus Strains of P72 Genotypes I and II in Domestic Pigs, Vietnam, 2023. Emerg. Infect. Dis..

[25] Chen S., Wang T., Luo R., Lu Z., Lan J., Sun Y., Fu Q., Qiu H.-J. (2024). Genetic Variations of African Swine Fever Virus: Major Challenges and Prospects. Viruses.

[26] Tilocca B., Greco V., Soggiu A., Urbani A., Britti D., Bonizzi L., Buonavoglia C., Roncada P. (2023). Multiepitope Array as the Key for African Swine Fever Diagnosis. Vet. Immunol. Immunopathol..

[27] Wang N., Zhao D., Wang J., Zhang Y., Wang M., Gao Y., Li F., Wang J., Bu Z., Rao Z. (2019). Architecture of African swine fever virus and implications for viral assembly. Science.

[28] Wang J., Bai J., Zhang L., Xia T., Yang X., Zhang K., Gao Y., Jiang P. (2023). A New B Cell Epitope of pC129R Protein of African Swine Fever Virus Identified by Monoclonal Antibodies. Vet. Microbiol..

[29] Jiang W., Jiang D., Li L., Wang J., Wang P., Shi X., Zhao Q., Liu B., Ji P., Zhang G. (2022). Identification of Two Novel Linear B Cell Epitopes on the CD2v Protein of African Swine Fever Virus Using Monoclonal Antibodies. Viruses.

[30] Tesfagaber W., Lan D., Wang W., Zhao R., Yin L., Yang M., Zhu Y., Sun E., Liu R., Lin W. (2024). Identification of Two Novel B Cell Epitopes on E184L Protein of African Swine Fever Virus Using Monoclonal Antibodies. Virus Res..

[31] Hu Y., Wang A., Yan W., Li J., Meng X., Chen L., Li S., Tong W., Kong N., Yu L. (2023). Identification of Linear Epitopes in the C-Terminal Region of ASFV P72 Protein. Microorganisms.

[32] Guo Y., Niu S., Wang X., Wang Z., Liang R., Tan Y., Fu Z., Su Z., Xu J., Chen H. (2025). ASFV pE146L-Induced ER Remodeling Is Essential for Viral Replication. J. Virol..

